# Systematic Review and Meta-Analysis of the Susceptibility of ABO Blood Groups to Venous Thromboembolism in Individuals with Factor V Leiden

**DOI:** 10.3390/diagnostics12081936

**Published:** 2022-08-11

**Authors:** Waleed M. Bawazir

**Affiliations:** 1 Medical Laboratory Technology Department, Faculty of Applied Medical Sciences, King Abdulaziz University, Jeddah 21589, Saudi Arabia; wbawazir@kau.edu.sa; Tel.: +966-560005541; 2 Hematology Research Unit, King Fahd Medical Research Center, King Abdulaziz University, Jeddah 21589, Saudi Arabia

**Keywords:** ABO blood group, factor V Leiden, meta-analysis, susceptibility, systematic review, venous thromboembolism

## Abstract

A limited number of studies investigated the association between the ABO blood groups and the incidence of venous thromboembolism in individuals with Factor V Leiden; however, discordant findings were reported. Consequently, this systematic review and meta-analysis aimed to evaluate the existing evidence on the susceptibility of the ABO blood group to venous thromboembolism in individuals with Factor V Leiden. All English-published articles on the Web of Science, Scopus, PubMed, EMBASE, and Google Scholar were comprehensively and systematically searched by the author without a time or region limit. Four studies were included in the qualitative synthesis and meta-analysis after the removal of studies that were not eligible. According to the analyses of the fixed and random effects, the point estimates of the effect size and the 95% confidence interval were 0.416 (95% CI: 0.397–0.435) and 0.392 (95% CI: 0.288–0.507), respectively. In contrast, the homogeneity test (Q value) reveals that blood group data distributions have a heterogenous structure (Q = 432.187; *p*-value < 0.001). The pooled event rates and the 95% CIs for the A, AB, B, and O-blood groups were 0.518 (95% CI: 0.411–0.622), 0.592 (95% CI: 0.495–0.683), 0.205 (95% CI: 0.041–0.612), and 0.283 (95% CI: 0.247–0.322), respectively. According to the findings, people with Factor V Leiden with blood group AB are more likely to develop venous thromboembolism than those with blood groups A, O, and B. The overall statistical significance of the ABO blood group’s susceptibility to venous thromboembolism in individuals with Factor V Leiden was <0.001 (pooled *p*-value). In conclusion, the current meta-analysis provides an additional indication that blood group AB individuals with Factor V Leiden are at higher risk of developing venous thromboembolism, and blood type B is connected to a lower risk of developing venous thromboembolism.

## 1. Introduction

One of the primary clinical symptoms of venous thromboembolism (VTE) is deep vein thrombosis (DVT) [1]. DVT is a multifactorial condition that results in hypercoagulation and is brought on by inherited and/or acquired defects of the hemostatic process [2]. Most cases of hereditary thrombophilia, also known as Factor V Leiden (FVL), Factor V: R506Q, or Factor V: G1691A, are responsible for predisposition to VTE, and the greatest prevalence is resistance to activated protein C. This resistance is most frequently caused by a point mutation in the coagulation factor V (FV) gene [3,4].

Furthermore, there is a great deal of disagreement surrounding the best way to manage carriers in terms of both the necessity and efficacy of antithrombotic therapies [5]. The relatively low risk of VTE associated with these polymorphisms and the awareness that VTE is a polygenic and complex disease are the causes of this confusion [6]. These polymorphisms should be viewed as thrombosis risk factors because the clinical manifestation of thrombosis typically depends on the coexistence of other thrombophilic mutations or environmental variables that cause the development of VTE [7].

The higher risk and greater severity of thrombosis in homozygous carriers, or people with combinations of several gene abnormalities, are examples of the impact of gene-gene interactions in VTE [8].

Activated protein C (APC)-resistance prevalence or FVL carrier frequency range from around 12 to 20 percent in patients who are primarily Caucasian and have experienced a first-ever venous thromboembolism, and they are linked to an increased risk of venous thromboembolism by 2.7 to 6.6 times [9,10,11]. The incidence of venous thromboembolism (also known as “absolute risk”) is more helpful for individual patient counseling, even while the degree of the elevated risk is valuable for identifying those factors that are most essential for potential change [12]. Uncertainty surrounds the prevalence of venous thromboembolism in FVL carriers. For carriers older than 15 years, reported crude incidence rates range from 280 to 670 per 100,000 person-years [13,14,15]. Additionally, the majority of research [13,14,15] focused on asymptomatic family members that were connected to symptomatic proband FV Leiden carriers.

The link between arterial thrombotic disorders and these frequent genetic variations is quite weak as the per-allele relative risks (RR) was 1.17 for FVL and 1.31 for PT, according to a meta-analysis including 66,155 cases and 91,307 controls) [16]. Many findings most likely reflect the extremely complicated nature of these illnesses, in which a single functional polymorphism’s impact is too modest to consistently correlate with risk.

It seems acceptable to assess the significance of gene-gene interactions on thrombotic risk within this paradigm. Unfortunately, there is not much research that has looked into this potential, especially in arterial thrombosis. Numerous investigations have conclusively shown that the ABO blood type has a clinically significant impact on in vivo hemostasis. Numerous studies have consistently shown that those with non-O blood had a much higher risk of VTE (between 1.8 and 2.5-fold) [17,18,19,20,21,22]. The ABO group has also been linked to increased risks of peripheral vascular disease and ischemic heart disease [23,24,25].

Patients with bleeding disorders, however, are overrepresented in the blood group O. It is interesting to note that FVL carriers with non-O blood groups appear to have a much higher risk of VTE. Unfortunately, because of the small numbers of carriers, particularly among controls, these findings were based on research with weak statistical power [26,27,28,29].

Since there is a better recognition of systematic review and meta-analysis findings in health policy and decision-making processes, this study was conducted to provide summary estimates on the relationship between the susceptibility of the ABO blood groups to venous thromboembolism in individuals with Factor V Leiden in order to reduce the gap in the literature and support evidence-based decision making.

## 2. Materials and Methods

### 2.1. Protocol Registration and PRISMA Statement

The protocol of the study was registered and approved by The International Prospective Register of Systematic Reviews (PROSPERO, registration No. CRD42022343973). In addition, the PRISMA recommendations were considered in this systematic review and meta-analysis (Supplemental Table S1).

### 2.2. Search Strategy for Literature and Data Sources

All English-published articles on the Web of Science, Scopus, PubMed, EMBASE, and Google Scholar were comprehensively and systematically searched by the author without a time or region limit. A combination of search methods was employed to increase the search scale, namely: (1) search MESH (medical subject heading) using the following terms: “ABO blood group”, “Venous Thromboembolism”, and “Factor V Leiden”, and (2) free-text search. Supplemental Table S2 presents the applied PubMed search strategy using MeSH terms and free texts as an example.

### 2.3. Eligibility Criteria (Inclusion/Exclusion)

The following were the inclusion requirements: Studies have described the frequency distribution of venous thromboembolism in people with factor V Leiden according to each blood group. On the other hand, the following exclusion criteria were considered: (1) Reviews; (2) Non-English language; (3) Case reports; (4) Studies where the end measure was not the frequency of venous thromboembolism in people with factor V Leiden in each blood type (A, AB, B, and O-blood group); (5) Studies lacking pertinent data; and (6) Studies without Full Text.

### 2.4. Study Screening and Data Extraction

The screening procedures were managed, and duplicates were eliminated using EndNote V.X8 software. After removing duplicates, the author individually screened the titles, abstracts, and full texts to determine whether the studies were eligible. The first author’s name, publication date, study title, nation of origin, gender, mean participant age, sample size, frequency, and percentage of venous thromboembolism in individuals with factor V Leiden cases according to each blood group category (A, AB, B, and O-blood group), as well as other information, were all extracted and recorded independently using a standardized data collection form that was developed in accordance with the sequence of variables required from the primary source.

### 2.5. Quality Assessment

The quality of the included studies was evaluated using the “The Quality Assessment Tool For Quantitative Studies (QATFQS),” which was developed by the “Effective Public Health Practice Project (EPHPP)” [30]. An assessment instrument was used since it is more thorough and enables a thorough evaluation of the included studies. Selection bias, study design, confounders, blinding, data collection methods, withdrawals and dropouts, intervention integrity, and analysis are the eight components of the assessment instrument that evaluate the study’s quality. Each component is scored individually in one of three categories (1 = Strong, 2 = Moderate, 3 = Weak), and for the overall study score: “1 = STRONG (no ratings of WEAK), 2 = MODERATE (one rating of WEAK), and 3 = WEAK” (two or more WEAK ratings) [31].

### 2.6. Data Synthesis and Statistical Analysis

All statistical analyses were done using Comprehensive Meta-Analysis Software (CMA, version 3, BioStat, Tampa, FL, USA). The number of studies that should be included in the meta-analysis to reset the effect size value obtained from the meta-analysis was estimated using the Fail-Safe N technique. The studies included in the meta-mean analysis’s effect sizes were calculated. The data is given in the forest plots. The ABO blood group susceptibilities to venous thromboembolism in people with Factor V Leiden were pooled and evaluated using a random-effect model. The retrieved data were used to calculate the event rate, their accompanying 95 percent confidence intervals, and the *p*-value.

The heterogeneity between the included studies was determined using the I^2^ statistic, and I^2^ values of 25%, 50%, and 75% are considered low, moderate, and high estimates, respectively [32]. When *p*-value < 0.1 or I^2^ < 50%, a non-significant level of statistical heterogeneity was assumed. The significant degree of variability led to the selection of a random-effect model [33]. A funnel plot was used to examine publication bias, and Begg’s and Mazumdar’s rank correlation tests were used to look for any indications of publication bias among the included papers.

## 3. Results

### 3.1. Search Results

**Figure 1:**
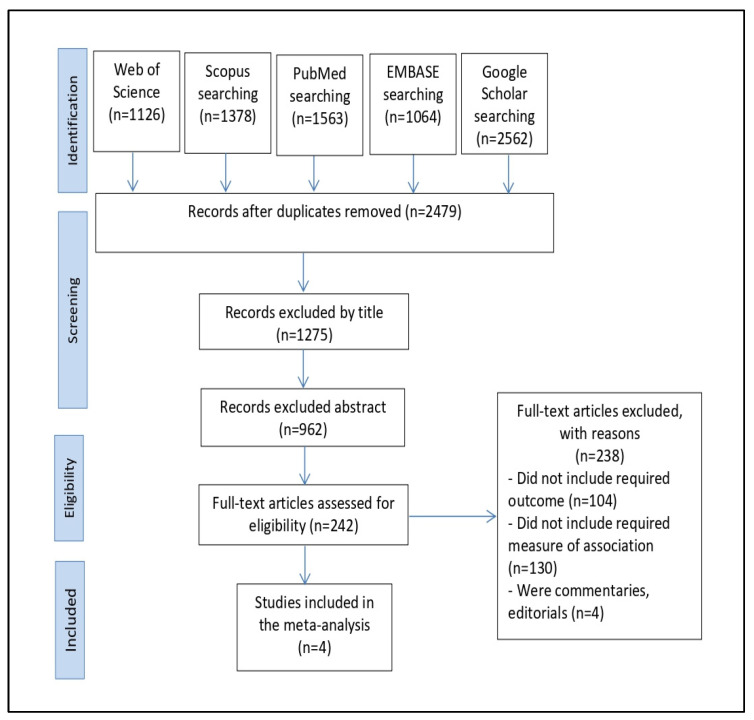
The PRISMA flowchart for the process of selecting and identifying studies.

In total, 7693 articles were found thanks to the search across four databases: Web of Science (*n* = 1126), Scopus (*n* = 1578), PubMed (*n* = 1563), EMBASE (*n* = 1064), Google Scholar (*n* = 2562). The duplicates were then removed, leaving 2479 studies. We eliminated 1275 studies by title screening and then used abstract screening to eliminate 962 irrelevant studies out of 1204. Following this, we read the full texts of the remaining 242 publications and omitted 238 pieces of research since they did not meet our inclusion criteria. The qualitative synthesis and meta-analysis eventually comprised four papers in total (Figure 1).

### 3.2. Characteristics of the Included Studies

The overall characteristics of the four studies [34,35,36,37] that satisfied our meta-analysis inclusion and exclusion criteria are shown in Table 1. The quality assessment for the included studies showed two studies were of strong quality, one was of moderate quality, and one was of poor quality. The poor-quality results were related to two items: (1) selection bias and (2) confounders (Supplemental Table S3). The included studies were conducted in 1999, 2004, 2008, and 2009. In addition, two studies were conducted in Spain, one in the Netherlands, and one in Brazil. Three studies used case-control in the design, and one was a cross-sectional study. The sample size of the studies ranged from 65 to 471 participants. The total number of participants in the four included studies was 792, of whom 163 were males and 158 were females; the study conducted in the Netherlands did not report the participants’ gender. Three studies reported the mean age of participants, which ranged between 34 and 56 years. The included studies mentioned the number of venous thromboembolisms in individuals with Factor V Leiden and the frequency distribution according to each blood group category (A, AB, B, and O-blood group); accordingly, we could calculate the percentages in each blood group.

Three studies in Spain and the Netherlands revealed that individuals with Factor V Leiden in the A blood group are at increased risk for venous thromboembolism with an A > O > B > AB distribution pattern. However, the study conducted in Brazil showed that individuals with Factor V Leiden in the AB blood group are at increased risk for venous thromboembolism with an AB > O > A = B distribution pattern (Table 1).

### 3.3. Integrated Outcomes

The fixed effect and random effect models are the two statistical models available for a meta-analysis. The fixed effect model presupposes that there is a single genuine effect size shared by all of the studies included in the meta-analysis and that any observed difference between studies is due to random chance or sampling mistakes [38]. The random effect model presupposes those studies differ significantly in a number of ways, and that the genuine impact size may change from one research to the next [33]. As a result, the random effect model evaluates both intra-study sampling mistakes and inter-study variance (between-study variation), whereas the fixed effect model only evaluates intra-study sampling errors (intra-study variation) [39]. As a result, the choice of meta-analysis model depends on whether heterogeneity is present or not. A fixed effect model is employed if there is no heterogeneity (heterogeneity *p* ≥ 0.10). However, a random effect model should be utilized for the meta-analysis when the Q-value is significant (*p* < 0.10), indicating that there is heterogeneity in the studies [40]. Both models produce comparable results when the study groups are homogeneous; however, when the study groups are heterogeneous, the random effect model frequently offers broader confidence intervals (CIs) than the fixed effect model [41].

According to the analyses of the fixed and random effects of the 16 subgroups within the 4 studies included, the point estimates of the effect size and the 95% confidence interval were 0.416 (95% CI: 0.397–0.435) and 0.392 (95% CI: 0.288–0.507), respectively. A point estimate should ideally be (1) Consistent. The estimate is more accurate the larger the sample size. (2) Neutral. The expectation of the average observation value, or “average observation value”, is equal to the associated population parameter. (3) The most effective or best unbiased estimate is the one with the smallest variance among all reliable, unbiased estimates (a measure of the amount of dispersion away from the estimate) [42]. In contrast, the homogeneity test (Q-value) reveals that blood group data distributions have a heterogenous structure (Q = 432.187; *p*-value < 0.001). The Q test, however, could not be accurate if there are only a few papers included in the meta-analysis. Given that Cochran’s Q test has poor statistical strength and is insensitive, a heterogeneity *p* value of < 0.10 (rather than 0.05) denotes the presence of heterogeneity [43].

We chose a random-effects model to carry out a meta-analysis in accordance with the sample’s heterogeneity. Thus, using the random-effects model, the relationship between the ABO blood group and the risk of venous thromboembolism in individuals with Factor V Leiden was investigated. The amount of real heterogeneity between the included studies (tau value) was 0.887.

For a fair assessment of the accuracy of meta-analyses, the produced homogeneity measure, I-squared (I^2^), evaluates the pooled fraction of variability between the included studies in a meta-analysis explained by differences rather than by sampling error [44]. The I^2^ values derived from the meta-analysis were lower than 99 percent (96.529%), indicating that there is a homogenous and narrow dispersion of effect sizes in the current meta-analysis (Table 2).

### 3.4. Orwin’s and Classic Fail-Safe N Findings

The number of studies that should be included in the meta-analysis to reset the effect size value obtained from the meta-analysis was estimated using the Fail-Safe N technique [45]. The effect value produced by our meta-analysis is relatively resistant to publication bias, as shown by the 286 N value, which was acquired by the standard Fail-Safe N method at a significantly high level (Table 3).

### 3.5. ABO Blood Group Susceptibility to Venous Thromboembolism in Individuals with Factor V Leiden

**Figure 2:**
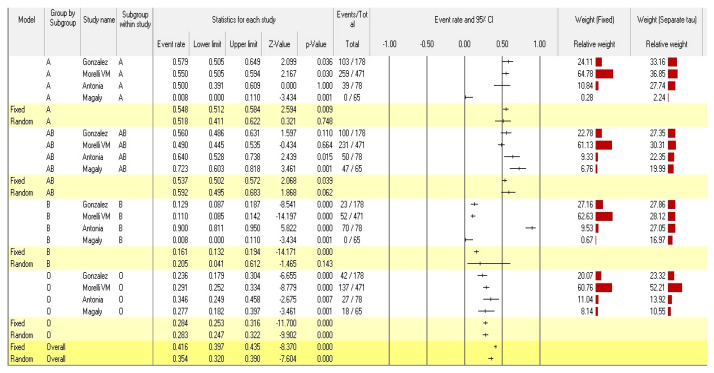
Forest plot from the fixed and random-effects analysis: ABO Blood group susceptibility to venous thromboembolism in individuals with Factor V Leiden [34,35,36,37].

The pooled event rates and the 95% CIs for the A, AB, B, and O-blood groups were 0.518 (95% CI: 0.411–0.622), 0.592 (95% CI: 0.495–0.683), 0.205 (95% CI: 0.041–0.612), and 0.283 (95% CI: 0.247–0.322), respectively. According to the findings, people with Factor V Leiden with blood group AB are more likely to develop venous thromboembolism than those with blood groups A, O, and B. The overall statistical significance of the ABO blood group’s susceptibility to venous thromboembolism in individuals with Factor V Leiden was <0.001 (pooled *p*-value) (Figure 2).

### 3.6. Rank Correlation

The findings of the rank correlation by Begg and Mazumdar test and Egger’s regression intercept showed no evidence of a substantial publication bias. The two-tailed *p*-values for Kendall’s tau with and without continuity and Egger’s regression intercept were 0.928, 0.964, and 0.661, respectively (Table 4).

### 3.7. Publication Bias

**Figure 3:**
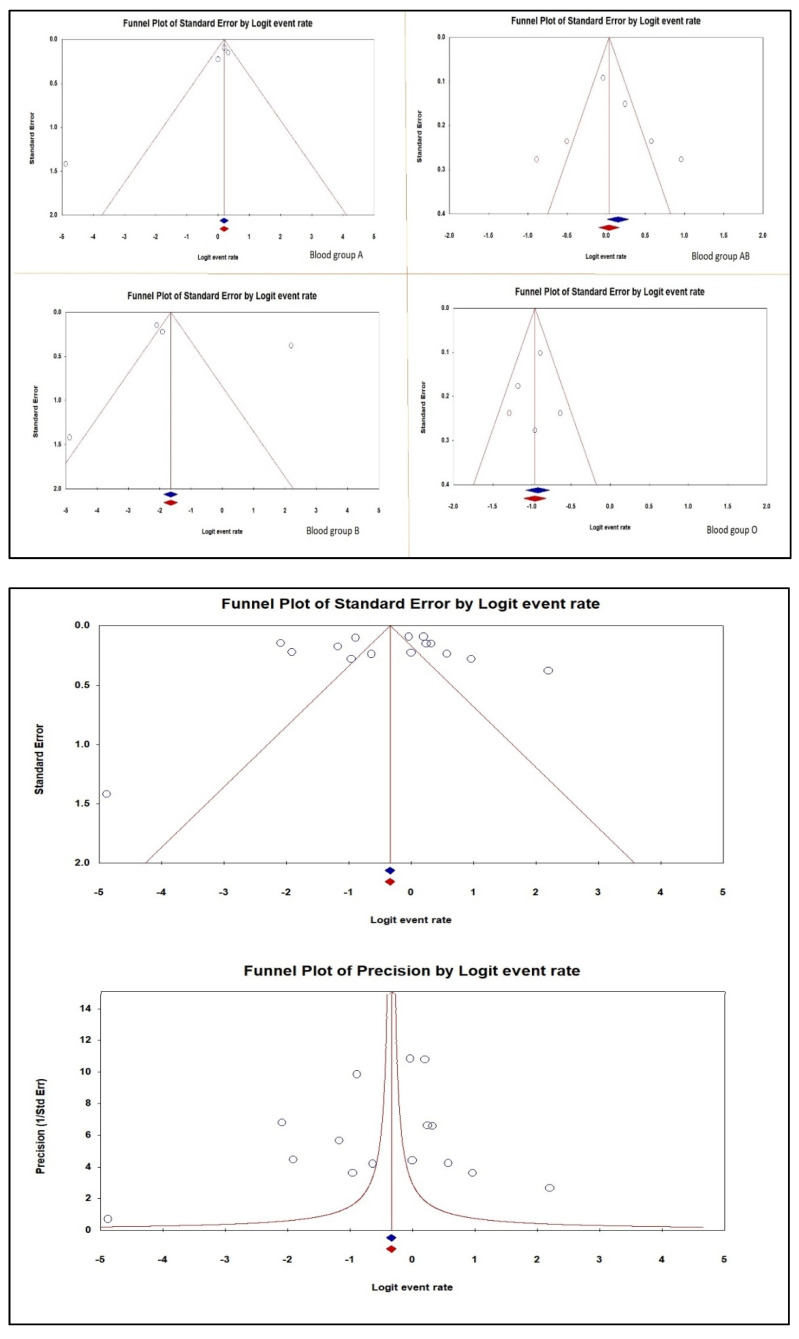
Publication bias of the ABO Blood group susceptibility to venous thromboembolism in individuals with Factor V Leiden [34,35,36,37].

Figure 3 displays the funnel plot of the ABO blood group susceptibility to venous thromboembolism in individuals with Factor V Leiden publication bias.

## 4. Discussion

Our meta-analysis found that the pooled event rates and the 95% CIs for the A, AB, B, and O-blood groups were 0.518 (95% CI: 0.411–0.622), 0.592 (95% CI: 0.495–0.683), 0.205 (95% CI: 0.041–0.612), and 0.283 (95% CI: 0.247–0.322), respectively. Accordingly, people with Factor V Leiden with blood group AB are more likely to develop venous thromboembolism than those with blood groups A, O, and B. However, in earlier research, it was discovered that non-O blood type increased the risk of venous thromboembolism. To answer this question, previous general population studies lacked the necessary power. The results of earlier investigations are supported by the cumulative effect of ABO blood type and factor V Leiden on the risk of venous thromboembolism [16,18,46].

The study conducted by Birgitte et al. revealed that prothrombin G20210A mutations and factor V Leiden R506Q accounted for 10% and 1%, respectively, of the population-attributable risk for venous thromboembolism and non-O blood type, respectively, for 20% of the population. This suggests that genetic testing for thrombophilia should include ABO blood type [47]. Their findings were in line with earlier research in that ABO blood type, factor V Leiden R506Q, and prothrombin G20210A mutations were not consistently linked to myocardial infarction [9,16,48].

In studies of thrombophilia, this prothrombotic genetic risk factor is typically not examined. However, considering that thrombosis is a multigenic and complex disease, the moderate thrombotic risk associated with the non-O blood group (OR: 1.8–2.5) and the high prevalence of prothrombotic non-O genotypes in the general population (>50 percent) make the ABO blood group of general interest in thrombosis [49]. It is possible to comprehend complex features by making the assumption that various candidate susceptibility gene mutations interact with one another. According to this model, individuals with the prothrombotic ABO genotype may be more susceptible to venous and arterial thromboses if they also carry additional prothrombotic risk factors.

Many studies have found a connection between various ABO blood types and a number of diverse, and often odd, phenotypic traits (including higher intelligence in group A2 individuals, increased criminality in group B individuals, and more severe hangovers in group A individuals) [50,51]. Many of these early association studies came to contradictory conclusions, and the tiny subject population further complicated the interpretation of the findings. As a result, all reported correlations with the ABO group were treated with considerable skepticism for a long time. This may help to understand the reason behind that skepticism despite a clear link between Factor V Leiden and the ABO blood group [18,19,21,37].

Since higher levels of von Willebrand factor and/or factor VIII are risk factors for venous thromboembolism, this link between ABO blood type and venous thromboembolism can be partially explained by the elevated amounts of these factors in non-O people’s blood [52]. The distribution of ABO antigens by von Willebrand factor on the membrane’s surface may result in decreased clearance of von Willebrand factor by ADAMST13 metalloproteinase [53].

Additionally, genetics account for 66 percent of the overall variation in plasma levels of von Willebrand factor, with ABO blood type accounting for 30 percent of this genetic component [54]. The observation of a shorter activated partial thromboplastin time in people with non-O blood type compared to O blood type is likely explained by the fact that people with non-O blood have 25–30% greater plasma levels of von Willebrand factor [52,55]. Although the association between von Willebrand factor levels and the increased risk of venous thromboembolism among people with non-O blood types has consistently been noted, recent findings from genome-wide association studies suggest that ABO antigens may also have an impact through additional mechanisms [56].

One of the factor V gene’s three activated protein C cleavage sites is eliminated by the single nucleotide mutation known as factor V Leiden R506Q. As a result, factor V is inactivated at a slower pace, increasing thrombin generation and venous thromboembolism risk [9]. Prothrombin G20210A is a single nucleotide mutation in the gene promoter’s untranslated region that results in increased prothrombin production [57], raising prothrombin levels by roughly 30% in heterozygous people and 70% in homozygous people [48]. The risk of venous thromboembolism ultimately increases as a result of this rise.

It would be helpful to understand how the ABO blood group interacts with other venous thrombosis risk factors, including genetic (antithrombin, protein C, or protein S deficits) and acquired risk factors (surgery, immobilization, pregnancy, oral contraceptives). VTE is a multicausal disease in which thrombosis must occur concurrently with a number of risk factors, both inherited and acquired. As a result, the majority of people who have a single thrombophilic risk factor, especially those who have common prothrombotic genotypes, are asymptomatic. Moreover, thrombophilia typically has no effect on the likelihood of recurrence or the length of anticoagulant medication.

Therefore, genetic testing for thrombophilia raises serious ethical questions regarding insurance and social security issues in asymptomatic carriers of single thrombophilic defects even though it is of limited value to the symptomatic patient and offers little benefit over and above the family history [58,59].

As a result, to more accurately assess a person’s risk of thromboembolic events, it is required to identify clusters of risk variables. Our findings indicate that those with Factor V Leiden who have an AB blood type have a considerably higher risk of venous thromboembolism. This suggests that ABO phenotyping or genotyping analyses may be useful tools for estimating future thrombophilic risk profiles and may have consequences for the management of thrombosis.

This systematic review and meta-analysis was the first study to examine the relationship between people with Factor V Leiden and the ABO blood group’s susceptibility to venous thromboembolism. However, the following restrictions should be taken into account while interpreting the current results: First off, only four eligible articles were included in this meta-analysis, and studies from different nations around the world were not included. As a result, we believe that those articles are still in the preliminary stages, and additional verification is required to guarantee their inherent quality. Second, despite our meta-strict analysis’s inclusion criteria, significant heterogeneity was discovered. Third, because adjusted confounders varied between trials, we were unable to do a subgroup analysis. Finally, we believe that the sample sizes used in the included studies were not accurate representations of the initial general populations.

## 5. Conclusions

We revealed that people with Factor V Leiden and blood type AB > A > O > B had a higher rate of venous thromboembolism. This evidence-based meta-analysis study also shows that blood group A people are more prone to venous thromboembolism. Venous thromboembolism risk is reportedly decreased in people with blood type B. However, the precise molecular and clinical mechanism underlying the variable sensitivity of ABO blood groups to venous thromboembolism in people with Factor V Leiden remains largely unknown. Therefore, more research is required to better understand how the ABO blood group affects venous thromboembolism and to determine whether stepping up infection control measures according to blood category could lower the chance of developing venous thromboembolism. Additionally, it is crucial to report blood types and subtypes for every infected person in every community so that they can later be correlated with a larger data set based on their demographic data, such as gender, age, ethnicity, etc.

## Figures and Tables

**Table 1:** The primary feature of the included studies [34,35,36,37].

The First Author (Year)	Country	Study Population	Study Design	Gender (M/F)	Mean Age	Sample n	O n (%)	A n (%)	B n (%)	AB n (%)	Prevalence
González Ordœñez et al. (1999)	Spain	Individuals with the factor V Leiden	Cross-sectional study	92/86	54	178	42 (23.6)	103 (57.9)	23 (12.9)	10 (5.6)	A > O > B > AB
Morelli VM et al. (2005)	Netherlands	Patients with venous thrombosis	Case-control study	NM	NM	471	137 (29.1)	259 (55.0)	52 (11.0)	23 (4.9)	A > O > B > AB
Antonia Miñano et al. (2008)	Spain	Carriers of factor V Leiden or prothrombin 20210A polymorphisms	Case-control study	53/25	56	78	27 (34.6)	39 (50.0)	7 (9.0)	5 (6.4)	A > O > B > AB
Magaly Lima et al. (2009)	Brazil	Patients had a history of DVT	Case-control study	18.47	34	65	18 (27.7)	0 (0.0)	0 (0.0)	47 (72.3)	AB > O > A = B

**Table 2:** The impact analysis values for the 16 subgroups within the 4 included studies in the meta-analysis, the homogenous distribution value, the average effect size, and the confidence intervals.

Model	Effect Size and 95% Confidence Interval				Test of Null (2-Tail		Heterogeneity				Tau-Squared			
Model	Number of Subgroups	Point of Estimate	Lower Limit	Upper Limit	Z-Value	*p*-Value	Q-Value	df (Q)	*p*-Value	I-Squared	Tau Squared	Standard Error	Variance	Tau
**Fixed**	16	0.416	0.397	0.435	−8.370	0.001	432.187	15	0.001	96.529	0.786	0.408	0.167	0.887
**Random**	16	0.392	0.288	0.507	−1.841	0.066								

**Table 3:** Orwin’s and Classic Fail-Safe N findings.

Orwin’s Fail-Safe N Method		Classic Fail-Safe N Method	
The event rate is observed in studies	0.416	Z-value for observed studies	−8.506
The criterion for a “trivial” event rate	0.500	The *p*-value for observed studies	0.001
Mean event rate in missing studies	0.500	Alpha	0.050
		Tails	2.000
		Z for alphas	1.959
		Number of observed subgroups in the studies	16.000
		Number of missing studies that would bring the *p*-value to > alpha (N value)	286.000

**Table 4:** Begg and Mazumdar rank correction and Egger’s regression intercept.

Kendall’s S Statistic (P-Q)	2.000
**Kendall’s tau without continuity correction**	
Tau	0.017
z-value for tau	0.090
*p*-value (1-tailed)	0.464
*p*-value (2-tailed)	0.928
**Kendall’s tau with continuity correction**	
Tau	0.008
z-value for tau	0.045
*p*-value (1-tailed)	0.482
*p*-value (2-tailed)	0.964
**Egger’s regression intercept**	
Intercept	−1.259
Standard error	2.807
95% low limit (2-tailed)	−7.280
95% upper limit (2-tailed)	4.762
t-value	0.448
df	14.000
*p*-value (1-tailed)	0.330
*p*-value (2-tailed)	0.661

## Data Availability

Not applicable.

## Supplementary Materials

The following supporting information can be downloaded at: https://www.mdpi.com/article/10.3390/diagnostics12081936/s1, Table S1: PRISMA recommendations checklist; Table S2: PubMed search strategy; Table S3: Quality assessment for the selected studies using The National Institute of Health quality assessment tool. Reference [60] is cited in Supplementary Materials.

## References

[1] Galanaud J.-P., Genty C., Sevestre M.-A., Brisot D., Lausecker M., Gillet J.-L., Rolland C., Righini M., Leftheriotis G., Bosson J.-L. (2011). Predictive factors for concurrent deep-vein thrombosis and symptomatic venous thromboembolic recurrence in case of superficial venous thrombosis. Thromb. Haemost..

[2] Smalberg J.H., Kruip M.J., Janssen H.L., Rijken D.C., Leebeek F.W., de Maat M.P. (2011). Hypercoagulability and hypofibrinolysis and risk of deep vein thrombosis and splanchnic vein thrombosis: Similarities and differences. Arterioscler. Thromb. Vasc. Biol..

[3] Rosendorff A., Dorfman D.M. (2007). Activated protein C resistance and factor V Leiden: A review. Arch. Pathol. Lab. Med..

[4] Bertina R.M., Koeleman B.P., Koster T., Rosendaal F.R., Dirven R.J., de Ronde H., van der Velden P.A., Reitsma P.H. (1994). Mutation in blood coagulation factor V associated with resistance to activated protein C. Nature.

[5] Mammen E.F. (2004). Current development in antithrombotic therapy. Semin. Thromb. Hemost..

[6] Benincasa G., Costa D., Infante T., Lucchese R., Donatelli F., Napoli C. (2019). Interplay between genetics and epigenetics in modulating the risk of venous thromboembolism: A new challenge for personalized therapy. Thromb. Res..

[7] Varga E., Kujovich J. (2012). Management of inherited thrombophilia: Guide for genetics professionals. Clin. Genet..

[8] Bucciarelli P., Rosendaal F.R., Tripodi A., Mannucci P.M., de Stefano V., Palareti G., Finazzi G., Baudo F., Quintavalla R. (1999). Risk of venous thromboembolism and clinical manifestations in carriers of antithrombin, protein C, protein S deficiency, or activated protein C resistance: A multicenter collaborative family study. Arterioscler. Thromb. Vasc. Biol..

[9] Ridker P.M., Hennekens C.H., Lindpaintner K., Stampfer M.J., Eisenberg P.R., Miletich J.P. (1995). Mutation in the gene coding for coagulation factor V and the risk of myocardial infarction, stroke, and venous thrombosis in apparently healthy men. N. Engl. J. Med..

[10] Koster T., Vandenbroucke J., Rosendaal F., de Ronde H., Briët E., Bertina R.M. (1993). Venous thrombosis due to poor anticoagulant response to activated protein C: Leiden Thrombophilia Study. Lancet.

[11] Folsom A.R., Cushman M., Tsai M.Y., Aleksic N., Heckbert S.R., Boland L.L., Tsai A.W., Yanez N.D., Rosamond W.D. (2002). A prospective study of venous thromboembolism in relation to factor V Leiden and related factors. Blood.

[12] Heit J.A., O’Fallon W.M., Petterson T.M., Lohse C.M., Silverstein M.D., Mohr D.N., Melton L.J. (2002). Relative impact of risk factors for deep vein thrombosis and pulmonary embolism: A population-based study. Arch. Intern. Med..

[13] Middeldorp S., Henkens C.M., Koopman M.M., van Pampus E.C., Hamulyak K., van der Meer J., Prins M.H., Buller H.R. (1998). The incidence of venous thromboembolism in family members of patients with factor V Leiden mutation and venous thrombosis. Ann. Intern. Med..

[14] Simioni P., Prandoni P., Girolami A. (1999). Low rate of venous thromboembolism in asymptomatic relatives of probands with factor V Leiden mutation. Ann. Intern. Med..

[15] Simioni P., Sanson B.-J., Prandoni P., Tormene D., Friederich P.W., Girolami B., Gavasso S., Huisman M.V., Büller H.R., ten Cate J.W. (1999). Incidence of venous thromboembolism in families with inherited thrombophilia. Thromb. Haemost..

[16] Ye Z., Liu E.H., Higgins J.P., Keavney B.D., Lowe G.D., Collins R., Danesh J. (2006). Seven haemostatic gene polymorphisms in coronary disease: Meta-analysis of 66 155 cases and 91 307 controls. Lancet.

[17] Jick H., Westerholm B., Vessey M., Lewis G., Slone D., Inman W.W., Shapiro S., Worcester J. (1969). Venous thromboembolic disease and ABO blood type: A cooperative study. Lancet.

[18] Ohira T., Cushman M., Tsai M., Zhang Y., Heckbert S., Zakai N., Rosamond W., Folsom A.R. (2007). ABO blood group, other risk factors and incidence of venous thromboembolism: The Longitudinal Investigation of Thromboembolism Etiology (LITE). J. Thromb. Haemost..

[19] Tirado I., Mateo J., Soria J.M., Oliver A., Martínez-Sánchez E., Vallvé C., Borrell M., Urrutia T., Fontcuberta J. (2005). The ABO blood group genotype and factor VIII levels as independent risk factors for venous thromboembolism. Thromb. Haemost..

[20] Streiff M.B., Segal J., Grossman S.A., Kickler T.S., Weir E.G. (2004). ABO blood group is a potent risk factor for venous thromboembolism in patients with malignant gliomas. Cancer.

[21] Larsen T.B., Johnsen S.P., Gislum M., Møller C., Larsen H., Sørensen H.T. (2005). ABO blood groups and risk of venous thromboembolism during pregnancy and the puerperium. A population-based, nested case-control study. J. Thromb. Haemost..

[22] Muellner S.K., Haut E.R., Streiff M.B., Holcomb J.B., Cotton B.A. (2011). ABO blood group as a potential risk factor for venous thromboembolism in acutely injured patients. Thromb. Haemost..

[23] Biswas J., Islam M., Rudra S., Haque M., Bhuiyan Z., Husain M., Mamun A. (2008). Relationship between blood groups and coronary artery disease. Mymensingh Med. J..

[24] Carpeggiani C., Coceani M., Landi P., Michelassi C., L’Abbate A. (2010). ABO blood group alleles: A risk factor for coronary artery disease. An angiographic study. Atherosclerosis.

[25] De Paula Sabino A., Ribeiro D.D., Domingheti C.P., Rios D.R.A., Dusse L.M.S., das Graças Carvalho M., Fernandes A.P. (2014). ABO blood group polymorphisms and risk for ischemic stroke and peripheral arterial disease. Mol. Biol. Rep..

[26] Franchini M., Favaloro E.J., Targher G., Lippi G. (2012). ABO blood group, hypercoagulability, and cardiovascular and cancer risk. Crit. Rev. Clin. Lab. Sci..

[27] Clark P., Wu O. (2011). ABO blood groups and thrombosis: A causal association, but is there value in screening?. Future Cardiol..

[28] Rosendaal F., Reitsma P. (2009). Genetics of venous thrombosis. J. Thromb. Haemost..

[29] Zhang H., Mooney C.J., Reilly M.P. (2012). ABO blood groups and cardiovascular diseases. Int. J. Vasc. Med..

[30] Armijo-Olivo S., Stiles C.R., Hagen N.A., Biondo P.D., Cummings G.G. (2012). Assessment of study quality for systematic reviews: A comparison of the Cochrane Collaboration Risk of Bias Tool and the Effective Public Health Practice Project Quality Assessment Tool: Methodological research. J. Eval. Clin. Pract..

[31] Thomas H., Ciliska D., Dobbins M. (2003). Quality Assessment Tool for Quantitative Studies.

[32] Higgins J.P., Thompson S.G. (2002). Quantifying heterogeneity in a meta-analysis. Stat. Med..

[33] DerSimonian R., Kacker R. (2007). Random-effects model for meta-analysis of clinical trials: An update. Contemp. Clin. Trials.

[34] Ordœñez A.G., Rodriguez J.M., Martin L., Alvarez V., Coto E. (1999). The O blood group protects against venous thromboembolism in individuals with the factor V Leiden but not the prothrombin (factor II G20210A) mutation. Blood Coagul. Fibrinolysis.

[35] Miñano A., Ordóñez A., España F., González-Porras J.R., Lecumberri R., Fontcuberta J., Llamas P., Marín F., Estellés A., Alberca I. (2008). AB0 blood group and risk of venous or arterial thrombosis in carriers of factor V Leiden or prothrombin G20210A polymorphisms. Haematologica.

[36] Lima M.B., Oliveira-Filho A.B.d., Campos J.F., Melo F.C., Neves W.B.d., Melo R.A.M., Lemos J.A.R. (2009). Increased risk of venous thrombosis by AB alleles of the ABO blood group and Factor V Leiden in a Brazilian population. Genet. Mol. Biol..

[37] Morelli V., de Visser M., Vos H., Bertina R., Rosendaal F. (2005). ABO blood group genotypes and the risk of venous thrombosis: Effect of factor V Leiden. J. Thromb. Haemost..

[38] Smith G.D., Egger M. (1997). Meta-analysis of randomised controlled trials. Lancet.

[39] Hedges L.V., Vevea J.L. (1998). Fixed-and random-effects models in meta-analysis. Psychol. Methods.

[40] Ried K. (2006). Interpreting and understanding meta-analysis graphs: A practical guide. Aust. Fam. Physician.

[41] Zintzaras E., Lau J. (2008). Synthesis of genetic association studies for pertinent gene–disease associations requires appropriate methodological and statistical approaches. J. Clin. Epidemiol..

[42] Lau J., Ioannidis J.P., Terrin N., Schmid C.H., Olkin I. (2006). The case of the misleading funnel plot. BMJ.

[43] Munafo M.R., Flint J. (2004). Meta-analysis of genetic association studies. Trends Genet..

[44] Borenstein M., Higgins J.P., Hedges L.V., Rothstein H.R. (2017). Basics of meta-analysis: I2 is not an absolute measure of heterogeneity. Res. Synth. Methods.

[45] Rothstein H.R., Sutton A.J., Borenstein M. (2005). Publication bias in meta-analysis. Publication Bias in Meta-Analysis: Prevention, Assessment and Adjustments.

[46] Weischer M., Juul K., Zacho J., Jensen G.B., Steffensen R., Schroeder T.V., Tybjærg-Hansen A., Nordestgaard B.G. (2010). Prothrombin and risk of venous thromboembolism, ischemic heart disease and ischemic cerebrovascular disease in the general population. Atherosclerosis.

[47] Sode B.F., Allin K.H., Dahl M., Gyntelberg F., Nordestgaard B.G. (2013). Risk of venous thromboembolism and myocardial infarction associated with factor V Leiden and prothrombin mutations and blood type. Can. Med Assoc. J..

[48] Ridker P.M., Hennekens C.H., Miletich J.P. (1999). G20210A mutation in prothrombin gene and risk of myocardial infarction, stroke, and venous thrombosis in a large cohort of US men. Circulation.

[49] Lane D.A., Grant P.J. (2000). Role of hemostatic gene polymorphisms in venous and arterial thrombotic disease. Blood.

[50] Garratty G. (2000). Blood groups and disease: A historical perspective. Transfus. Med. Rev..

[51] Greenwell P. (1997). Blood group antigens: Molecules seeking a function?. Glycoconj. J..

[52] Shima M., Fujimura Y., Nishiyama T., Tsujiuchi T., Narita N., Matsui T., Titani K., Katayama M., Yamamoto F.i., Yoshioka A. (1995). ABO blood group genotype and plasma von Willebrand factor in normal individuals. Vox Sang..

[53] Oriol R., Mollicone R., Coullin P., Dalix A.-M., Candelier J.-J. (1992). Genetic regulation of the expression of ABH and Lewis antigens in tissues. APMIS. Suppl..

[54] O’Donnell J., Boulton F.E., Manning R.A., Laffan M.A. (2002). Amount of H antigen expressed on circulating von Willebrand factor is modified by ABO blood group genotype and is a major determinant of plasma von Willebrand factor antigen levels. Arterioscler. Thromb. Vasc. Biol..

[55] Orstavik K., Magnus P., Reisner H., Berg K., Graham J., Nance W. (1985). Factor VIII and factor IX in a twin population. Evidence for a major effect of ABO locus on factor VIII level. Am. J. Hum. Genet..

[56] Wu O., Bayoumi N., Vickers M., Clark P. (2008). ABO (H) blood groups and vascular disease: A systematic review and meta-analysis. J. Thromb. Haemost..

[57] Franco R., Trip M., Ten Cate H., van den Ende A., Prins M., Kastelein J., Reitsma P. (1999). The 20210 G→ A mutation in the 3′-untranslated region of the prothrombin gene and the risk for arterial thrombotic disease. Br. J. Haematol..

[58] Bauer K.A. (2004). Role of thrombophilia in deciding on the duration of anticoagulation. Semin. Thromb. Hemost..

[59] Lochhead P., Miedzybrodzka Z. (2007). The essential role of genetic counseling in inherited thrombophilia. Semin. Hematol..

[60] Moher D., Liberati A., Tetzlaff J., Altman D.G., The PRISMA Group (2009). Preferred Reporting Items for Systematic Reviews and Meta-Analyses: The PRISMA Statement. PLoS Med..

